# Fatal Acute Hemorrhagic Encephalomyelitis and Antiphospholipid Antibodies following SARS-CoV-2 Vaccination: A Case Report

**DOI:** 10.3390/vaccines10122046

**Published:** 2022-11-30

**Authors:** Annika Kits, Mattia Russel Pantalone, Christopher Illies, Aleksandra Antovic, Anne-Marie Landtblom, Ellen Iacobaeus

**Affiliations:** 1 Department of Neuroradiology, Karolinska University Hospital, 171 76 Stockholm, Sweden; 2 Department of Clinical Neuroscience, Division of Neurology, Karolinska Institute and Neurology, Karolinska University Hospital, 171 76 Stockholm, Sweden; 3 Karolinska University Laboratory, Department of Clinical Pathology and Cancerdiagnostics, Karolinska University Hospital, 171 76 Stockholm, Sweden; 4 Department of Medicine, Division of Rheumatology, Karolinska Institute and Rheumatology, Karolinska University Hospital, 171 76 Stockholm, Sweden; 5 Department of Medical Sciences, Division of Neurology, Uppsala University, 751 85 Uppsala, Sweden; 6 Department of Biomedical and Clinical Sciences, Linköping University, 581 83 Linkoping, Sweden

**Keywords:** COVID-19, vaccination, acute hemorrhagic encephalomyelitis, antiphospholipid syndrome, antiphospholipid antibodies, adverse event

## Abstract

Acute hemorrhagic encephalomyelitis (AHEM) is a rare hyperacute form of acute disseminated encephalomyelitis (ADEM). The disease is characterized by fulminant inflammation and demyelination in the brain and spinal cord and is often preceded by an infection or vaccination. This case report presents a 53-year-old male with rheumatoid arthritis and ongoing treatment with methotrexate and etanercept who developed fatal AHEM following the second dose of the COVID-19 vaccine. The disease course was complicated by multiorgan thromboembolic disease and the presence of high/moderate levels of cardiolipin IgG antibodies and anti-beta-2 glycoprotein 1 IgG antibodies suggesting a possible antiphospholipid syndrome. Treatment with immunosuppressive therapies failed to improve the course. The report comprises comprehensive clinical, neuroimaging, and neuropathological findings. The case highlights diagnostic challenges in a patient with several preceding risk factors, including autoimmune disease, immunotherapy, and vaccination, with possible pathophysiological implications. The temporal association with the COVID-19 vaccination may suggest possible causality although evidence cannot be ascertained. Reporting possible adverse events following COVID-19 vaccination is important to identify at-risk populations and to accomplish control of the current pandemic.

## 1. Introduction

Acute hemorrhagic encephalomyelitis (AHEM), also termed acute hemorrhagic leukoencephalitis (AHLE/AHL) or Weston–Hurts Syndrome, is a rare, often lethal, hemorrhagic variant of acute disseminated encephalomyelitis (ADEM). The pathogenic mechanisms are considered to involve immune-mediated blood vessel injuries, inflammation, perivascular demyelination, hemorrhage, and necrosis [1]. Rapid neurological deterioration and mortality rates reaching 70% are typical in AHEM and contrast with the more favorable prognosis of ADEM [1]. AHEM, as well as ADEM, is frequently associated with an existing or previous infection or vaccination although post-vaccination ADEM is rare and represents only 5–10% of ADEM cases [2]. Several reports of ADEM following COVID-19 vaccination have recently been described while the presence of AHEM phenotypes occurs only rarely where reported [3]. Further, a wide range of neurological symptoms have been described in persons infected with COVID-19 in addition to central nervous system (CNS) hemorrhagic vascular injuries, inflammation, and demyelination with similar features to ADEM and AHEM [4].

COVID-19 has been assigned as a prothrombotic disease with hypercoagulability and endothelial cell injury, associated with inflammation, as key pathogenic factors [5]. Thromboembolic events commonly occur in COVID-19 patients and a high prevalence of antiphospholipid antibodies (aPL) was detected in critically ill patients [6]. Interestingly, a recent COVID-19 cohort study showed that the presence of anti-phosphatidylserine/prothrombin (aPS/PT) IgG was correlated with neurological manifestations [7]. Antiphospholipid syndrome (APS) is an autoimmune multiorgan disease associated with recurrent thromboembolic events, thrombocytopenia, and fetal loss, in addition to cardiac, dermatological, and neurological manifestations and persisting aPL antibodies [8]. A population-based study estimated the incidence of APS as 2.1/100,000 and the prevalence as 50/100,000 in the general adult population [9]. The presence of aPL antibodies is more frequent among patients with autoimmune diseases, infections, malignancies, and systemic inflammatory diseases. A wide range of CNS manifestations including cerebrovascular disease and demyelinating disease have been linked to both APS and the presence of aPL antibodies, and APS is an important differential diagnosis to consider for patients with symptoms and signs of CNS inflammatory disease [10].

In this case study, we report a patient that presented with AHEM, systemic venous thromboembolism, and subsequent presence of moderate/high levels of aPL antibodies, indicating a possible APS, following the second Pfizer-BioNTech COVID-19 vaccination. The case illustrates the diagnostic and therapeutic challenges in a patient with fulminant progression of CNS inflammation and thromboembolism and complicating preceding autoimmune disease, immunosuppressive treatment and vaccination.

## 2. Case Description

**Figure 1:**
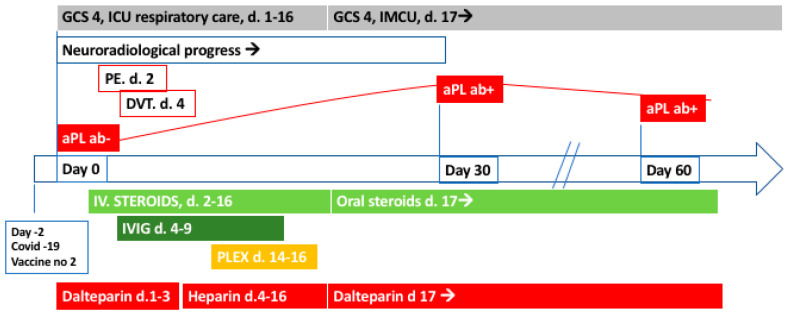
Schematic illustration of the clinical course and management of the patient. GCS; Glasgow coma scale, ICU; intensive care unit, IMCU: intermediate care unit, PE; pulmonary embolism, DVT; deep vein thrombosis, aPL ab; antiphospholipid antibodies, d.; day. IVIG = intravenous immunoglobulin therapy, PLEX; plasma exchange therapy.

**Figure 2:**
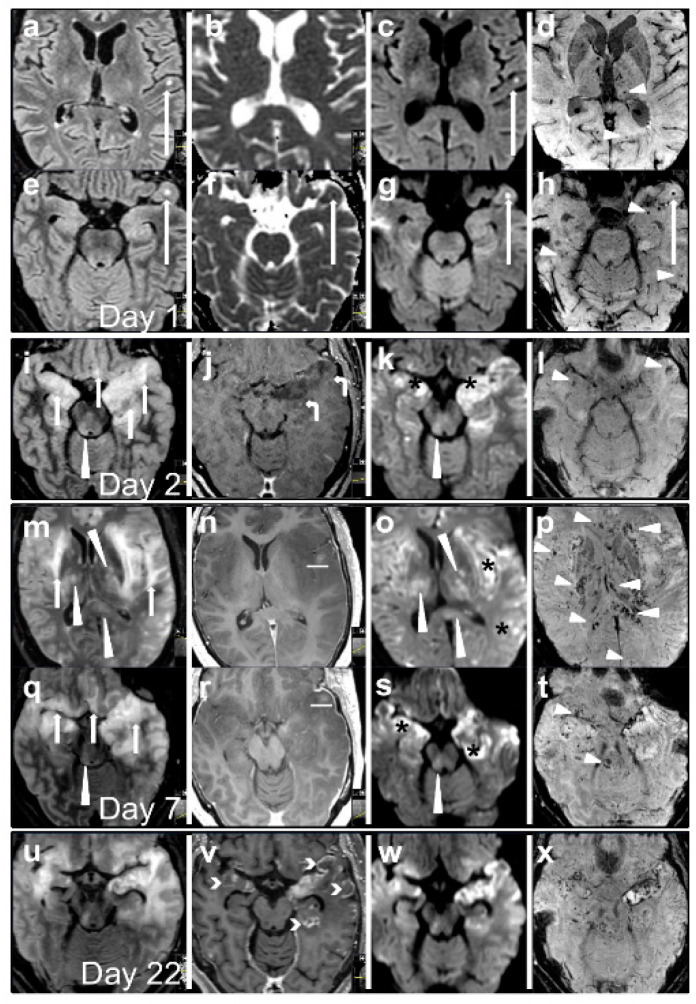
Axial MRI illustrating the evolution of brain lesions from day 1 (**a**–**h**), 2 (**i**–**l**), and 7 (**m**–**t**) to 22 (**u**–**x**) at the level of temporal lobes (**e**–**l**,**q**–**x**) and the level of the insula and basal ganglia (**a**–**d**,**m**–**p**) on fluid-attenuated inversion recovery (FLAIR) (**a**,**e**,**i**,**m**,**q**,**u**), apparent diffusion coefficient (ADC) (**b**,**f**), diffusion-weighted (DWI) (**c**,**g**,**k**,**o**,**s**,**w**), susceptibility-weighted (SWI) and T1-weighted contrast-enhanced images (**j**,**n**,**r**,**v**). Focal lesions on the first day (long arrow heads in (**a**–**h**)). Progression of extensive confluent areas of striking FLAIR hyperintensity (short arrow heads in (**i**,**m**,**q**)) in temporal, frontobasal, and insular regions as well as moderate FLAIR hyperintensity (long arrow heads in (**i**,**k**,**m**,**o**,**q**,**s**)) in basal ganglia, brainstem and corpus callosum, with the marked progression of petechial hemorrhages on SWI (small arrowheads, (**d**,**h**,**l**,**p**,**t**)), restricted diffusion on DWI (asterisk in (**k**,**o**,**s**)), leptomeningeal contrast-enhancement (curved arrow heads in (**j**)) and hemorrhagic transformation (horizontal white line in (**n**,**r**)). On day 22 contrast enhancement with cortical laminar necrosis appeared (brackets in (**v**)) and swelling started to reduce (widening of sulci and temporal horns of the lateral ventricles in (**u**–**w**) compared to (**q**–**s**)).

A 53-year-old man developed confusion and unconsciousness over a few hours and presented at the emergency room with a Glascow Coma Scale of 7, agitation, and snoring. He had received his second injection of Pfizer-BioNTech COVID-19 vaccine two days earlier (Figure 1). Neurological examination revealed anisocoria, right side miosis, bilateral absence of pupillary light reflex, and reduced voluntary movements in the left arm and leg. The patient was afebrile without clinical or laboratory signs of infection. He was intubated and admitted to the intensive care unit (ICU). Previous medical history included rheumatoid arthritis (RA) with ongoing treatment with etanercept and methotrexate. Acute brain computed tomography (CT) demonstrated a small hypoattenuating area in the left temporal lobe interpreted as an acute/subacute ischemic lesion, reduced perfusion in the left hemisphere, and normal CT angiography. Magnetic resonance imaging (MRI) demonstrated multiple cortical and subcortical lesions with high T2 and FLAIR signal and ubiquitous petechial hemorrhages (Figure 2). Pronounced neuroradiological progress was found on repeated MRI scans with the development of widespread lesions in cortical gray matter, thalami, basal ganglia, corpus callosum, brainstem, and cerebellum. Multiple lesions, mostly in the gray matter, were detected in the cervical and thoracic medulla. After three weeks, cortical laminar necrosis, a decrease in brain swelling, and regional encephalomalacia appeared in addition to increasing bilateral confluent lesions with increased signal in FLAIR and DWI, possibly representing demyelination that was delayed.

Initial cerebrospinal fluid (CSF) analysis showed elevated albumin (736 mg/L, ref < 400 mg/L) and CSF/serum-albumin ratio: 20.4 (ref < 9) but normal cell count. Repeated CSF analyses demonstrated pleocytosis: 58 cells × 10^6^/L (granulocytic dominance), normal CSF-IgG index and absence of CSF-specific oligoclonal IgG bands (OCB) at day 4 and increased neurofilament light chain levels: >100,000 ng/L (ref: <890 ng/L) and interleukin 6.25 ng/L (ref < 6 ng/L) at day 20. Comprehensive serum and CSF microbiological screening were negative for active infection including bacterial and fungal growth cultures, PCR for enterovirus, herpes simplex virus (HSV) 1 and 2, varicella-zoster virus, herpes virus type 6, Epstein–Barr virus, toxoplasma gondii, haemophilus influenza, listeria monocytogenes, Neisseria meningitides, streptococcus agalactiae, streptococcus pneumonia, mycobacterium tuberculosis and serology for borrelia burgdorferi, tick-borne encephalitis, cryptococcus, HIV-1 and 2, syphilis, nasopharyngeal PCR of SARS-CoV-2, influenza-A, and B and RS virus. The patient was seropositive for SARS-CoV-2 spike IgG (160 U/mL), interpreted as an immune response after the first COVID-19 vaccination dose. The patient had no history of symptomatic COVID-19 infection and the first COVID-19 vaccination had not caused adverse symptoms. Analyses for neuronal antibodies (CSF and serum), anti-MOG IgG, anti-aquaporin-4 IgG, and ANA screening were negative. Initial screening for aPL antibodies showed normal levels but repeated analyses at day 30 demonstrated high levels of anti-cardiolipin IgG antibodies (aCL): 99 E/mL (ref. < 10 E/mL) and anti-beta-2 glycoprotein 1 (aB2GP1) IgG antibodies: 60 E/mL (ref: <30 E/mL) with subsequent decline at day 60 (aCL IgG: 16 E/mL and aB2GP1 IgG: 9 E/mL). Analyses of aCL IgM, aB2GP1 IgM, and lupus anticoagulant were negative. Further coagulation screening showed normal levels of platelet count, activated partial thromboplastin time (APTT), international normalized ratio (INR), protein C, protein S, and normal factor II and factor V Leiden genotypes but elevated p-fibrin-D-dimer: 2.9 mg/L FEU (ref < 0.52 mg/L FEU) and p-fibrinogen: 4, 3 (ref: 2–4.2) at initial analysis (day 2). Assessment of a possible malignancy disorder with whole body FDG-PET CT scan, CSF cytology, and analyses of paraneoplastic antibodies in CSF and serum ruled out such condition.

**Figure 3:**
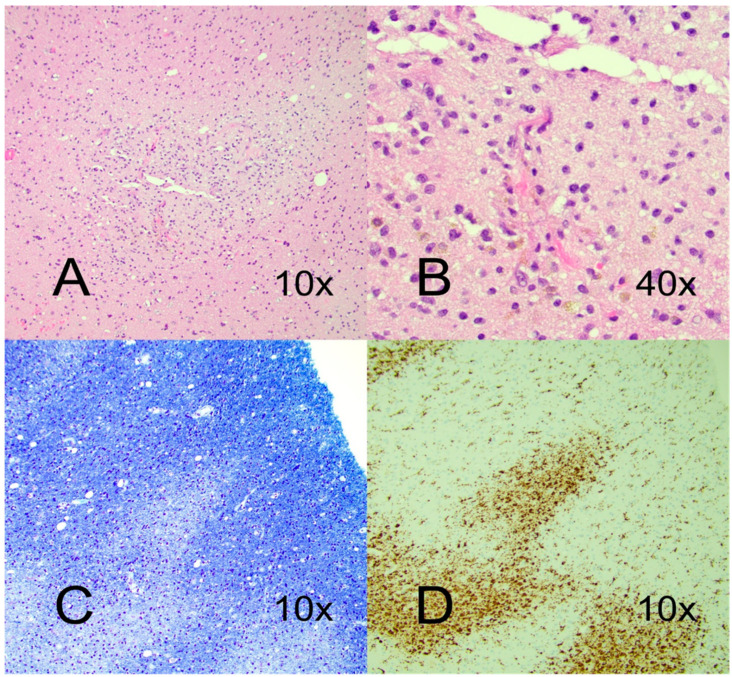
Hematoxylin and eosin (HE)—stained section from right temporal lobe biopsy demonstrated perivascular infiltrates of macrophages and small CD3+ lymphocytes (**A**) with hemosiderin deposits as a sign of former hemorrhage (**B**). Luxol fast blue stain showed perivascular demyelination (**C**), and CD68+ stained macrophage infiltrates (**D**).

The initially suspected diagnosis was ischemic stroke and the patient received intravenous (i.v.) thrombolysis administered 3.5 h after symptom onset. The diagnosis was re-evaluated following diagnostic work-up and treatment with acyclovir and high dose i.v. steroids (betamethasone 32 mg/day, day 2–4, followed by methylprednisolone, 1 g/day for 5 days, two cycles, during day 5–16) were administrated, with the suspicion of encephalitis or ADEM. Further, the patient received i.v. IgG therapy (2 g/kg/day) for five days starting from day four and three plasma exchange sessions from day fourteen. Oral steroids (prednisolone 50 mg/day with slow tapering) were administrated from day seventeen. The patient developed bilateral pulmonary embolism (day two) and venous brachial thrombosis (day four) despite treatment with full dose dalteparin which therefore was converted to full dose Heparin. The patient received dialysis for acute renal failure for one week during the ICU stay. His neurological condition did not improve but after stabilization of vital functions, he was transferred to an intermediate care unit on day 17. Due to the lack of clinical improvement and unresponsiveness to conventional immunosuppressive regimen, in addition to the continuous neuroradiologically visualized deterioration, a brain biopsy from the right temporal lobe was performed one month after symptom onset. Neuropathological examination showed white matter perivascular and partially confluent demyelinated areas with infiltrates of CD68+ macrophages and scattered CD3+T-cells (Figure 3). Perivascular hemosiderin deposits were found as a sign of previous hemorrhage. Surrounding tissue demonstrated reactive gliosis. The neuropathological observations and the MRI findings which both demonstrated inflammation, demyelination, micro-thromboembolisms, and microhemorrhage were consistent with a diagnosis of AHEM. The patient remained in a persistent vegetative status and died around four months after symptom onset, following palliative care referral.

## 3. Discussion

Diagnostic criteria for AHEM have not been defined but the present case fulfills the criteria of ADEM [2,11]. The postulated differential diagnoses were ischemic stroke or vasculitis with hemorrhagic transformation, infectious meningoencephalitis, autoimmune encephalitis and COVID-19-related disease, besides ADEM/AHEM. The initial MRI findings resembled those associated with HSV encephalitis, but similar pathologies have also been found in AHEM cases [12]. The neuroimaging development and the negative microbiological screening ruled out HSV. Additional early differential diagnoses were embolic infarcts or primary CNS vasculitis due to the focal cortical lesions with diffusion restriction. However, the flocculus lesions and spinal cord lesions were unlikely locations for ischemic infarctions and this diagnosis was considered unlikely. Further, the subsequent progressive enlargement of brain lesions and the normal CT angiography were not consistent with vasculitis [13]. COVID-19 associated encephalitis was also considered a possible diagnosis as it has been associated with both neuroimaging and neuropathological findings similar to AHEM [4]. However, the clinical course, PCR tests, and thoracic CT examination showed absence of findings consistent with COVID-19.

The detection of medium/high titers of aCL IgG and aB2GP1 IgG antibodies in addition to multiple venous thromboses with pulmonary, CNS, and deep vein affection raised suspicion of APS or catastrophic APS (CAPS). Initiation with more aggressive immunotherapy, such as rituximab or eculizumab was discussed but never initiated due to clinical stabilization. A definite APS was not confirmed as follow-up analyses of aPL antibodies were missing. The patient had no history of a prior APS and assessment of aPL antibodies had previously not been performed.

Infections, systemic inflammation, and vaccinations may trigger de novo synthesis of aPL through molecular mimicry mechanisms. However, a recent study reported only occasionally increased aPL antibodies in low titers after vaccination of healthy individuals with both RNA- and adenovirus-based COVID-19 vaccines [14]. Further, there were no vaccine-related new thrombotic events or APS flares observed in a cohort of APS patients [15]. It may be suggested that the transient rise in aPL antibodies in our patient reflected bystander pathogenic autoimmune mechanisms associated with endothelial cell injury and exposure to self-antigens rather than a primary APS. Nevertheless, the presence of aPL antibodies likely had clinical and pathogenic significance for the fulminant course with extensive CNS microvascular affection. Additionally, the presentation of aPL antibodies implicated enhanced surveillance for the development of additional thromboembolic events and optimization of anticoagulant strategies.

Interestingly, the present case had several similarities with three COVID-19 vaccine-associated AHEM cases recently described in a report from Germany [3]. In line with the present case, rapid severe clinical deterioration and coma, neuroimaging findings of bilateral white and grey matter supratentorial lesions with hemorrhagic involvement, absence of CSF-OCB, and poor outcome characterized these patients [3]. Contrasting our case, these three patients presented symptoms following their first COVID-19 injection while the present case had symptom onset after his second injection. In agreement with the three German cases, ADEM has mainly been described as a “first vaccine event”. It is possible that the treatment with methotrexate and etanercept, in our patient, only triggered a weak immune response to the first COVID-19 vaccine injection while the second injection caused a stronger vaccine response. Reduced antibody responses after COVID-19 vaccination have been observed in patients with chronic inflammatory disease and treatment with tumor necrosis factor-a inhibitors [16]. The absence of aPL antibodies was reported in 1/3 of the German AHEM cases while no information about aPL antibody testing or results in the two other patients was given. Previous case reports on COVID-19 vaccine-associated ADEM have occasionally described negative aPL antibodies but information about testing and testing results are in general limited. To the best of our knowledge, AHEM and possible APS following COVID-19 vaccination has previously not been reported in the literature.

The mechanisms associated with vaccine-induced ADEM have not been clarified but autoimmune reactions involving molecular mimicry between components of the vaccine and endogenous CNS structures have been suggested to play a role. Molecular mimicry seems to be involved in COVID-19 induced Guillain–Barré syndrome through anti-ganglioside antibodies supporting similar pathophysiological mechanisms in CNS demyelinating diseases [17]. There are also considerations regarding mRNA vaccines that may trigger a type 1 IFN response and aPL antibody production leading to the thrombotic manifestations associated even to CAPS [18,19,20]. A few cases of CAPS onset shortly after the first dose of mRNA COVID-19 vaccine have been described and it was speculated that the vaccination might have acted as a “second-hit” triggering CAPS in persons with aPL positive serological background [19,20].

## 4. Conclusions

AHEM is a life-threatening condition with diagnostic difficulties for which early recognition and treatment initiation is crucial to improve prognosis. The current case with detection of aPL antibodies and concomitant multiple thromboembolic events point out the importance of testing for a possible APS and aPL antibodies in patients with severe CNS neuroinflammatory conditions. High surveillance for the occurrence of thromboembolic events and early consultation with coagulation specialists are emphasized.

The temporal correlation with COVID-19 vaccination along with previous reports of ADEM onset after COVID-19 vaccination, may suggest a possible causal relationship although our report does not provide evidence for this.

Reporting possible side effects following COVID-19 vaccination is important to increase the control of the current COVID-19 pandemic and to identify risk groups for vaccine-induced adverse events. Immune compromised persons, like the current patient with an underlying autoimmune disease and immunosuppressive therapy, constitute such risk groups, but may as well have an increased risk for worse outcome of infection. Observational longitudinal studies are warranted to assess a possible causal link between COVID-19 vaccination and ADEM/AHEM and to enhance the knowledge about risk groups for vaccination and infection, specifically when the latter eventually tends to be milder.

## Data Availability

All of the relevant data are provided in the paper.
